# Prevalence of symbionts and trypanosome infections in tsetse flies of two villages of the “Faro and Déo” division of the Adamawa region of Cameroon

**DOI:** 10.1186/s12866-018-1286-5

**Published:** 2018-11-23

**Authors:** Ginette Irma Kame-Ngasse, Flobert Njiokou, Tito Trésor Melachio-Tanekou, Oumarou Farikou, Gustave Simo, Anne Geiger

**Affiliations:** 1 Laboratory of Parasitology and Ecology, Faculty of Science, Department of Animal Biology and Physiology, University of Yaoundé 1, Yaoundé, Cameroon; 2 Ministry of livestock, Fisheries and Animal Industries, Special Mission for Tsetse Eradication (MSEG), Ngaoundéré, Cameroon; 3 Molecular Parasitology and Entomology Unit, Faculty of Science, Department of Biochemistry, University of Dschang, Dschang, Cameroon; 4 Institut de Recherche pour le Développement (IRD)-CIRAD, UMR 177, Montpellier, France

**Keywords:** Animal African trypanosomiasis, *Trypanosoma* spp., Symbionts, Tsetse flies, Tripartite interactions, “Faro and Déo” division, Cameroon

## Abstract

**Background:**

Tsetse flies are vectors of human and animal African trypanosomiasis. In spite of many decades of chemotherapy and vector control, the disease has not been eradicated. Other methods like the transformation of tsetse fly symbionts to render the fly refractory to trypanosome infection are being evaluated. The aim of the present study was to evaluate the association between trypanosome infections and the presence of symbionts in these tsetse species. Tsetse flies were trapped in two villages of the “Faro and Déo” Division of the Adamawa region of Cameroon. In the field, tsetse fly species were identified and their infection by trypanosomes was checked by microscopy. In the laboratory, DNA was extracted from their midguts and the presence of symbionts (*Sodalis glossinidius* and *Wolbachia* sp.) and trypanosomes was checked by PCR. Symbionts/trypanosomes association tests were performed.

**Results:**

Three tsetse fly species including *Glossina tachinoides* (90.1%), *Glossina morsitans submorsitans* (9.4%) and *Glossina fuscipes fuscipes* (0.5%) were caught. In all the population we obtained an occurrence rate of 37.2% for *Sodalis glossinidius* and 67.6% for *Wolbachia* irrespective to tsetse flies species. *S. glossinidius* and *Wolbachia* sp. occurrence rates were respectively 37 and 68% for *G. tachinoides* and 28.6 and 59.5% for *G. m. submorsitans*. Between Golde Bourle and Mayo Dagoum significant differences were observed in the prevalence of symbionts. Prevalence of trypanosomes were 34.8% for *Glossina tachinoides* and 40.5% for *Glossina morsitans submorsitans.* In *G. tachinoides,* the trypanosome infection rates were 11, 2.6 and 13.7%, respectively, for *T. brucei* s.l., *T. congolense* forest type and *T. congolense* savannah type. In *G. m. submorsitans,* these infection rates were 16.7, 9.5 and, 2.4% respectively, for *T. brucei* s.l.*, T. congolense* forest type and *T. congolense* savannah type.

**Conclusions:**

The rate of tsetse fly infection by trypanosomes was low compared to those obtained in HAT foci of south Cameroon, and this rate was not statistically linked to the rate of symbiont occurrence. This study allowed to show for the first time the presence of *Wolbachia* sp. in the tsetse fly sub-species *Glossina morsitans submorsitans and Glossina tachinoides*.

## Background

Tsetse flies are vectors of parasitic diseases, namely, sleeping sickness or Human African Trypanosomiasis (HAT) in humans and nagana or Animal African Trypanosomiasis (AAT) in cattle. Nagana remains one of major obstacles constraining livestock development in several sub-Saharan African regions. It is responsible for the death of about 3 million cattle every year, the loss of 26% of dairy yield and the reduction of 50% of the number of cattle herds used in high potential agricultural areas [1]. In addition, the shortfall in agriculture due to nagana is estimated at 4.5 billion US$ per year [2]. AAT constitutes therefore one of the major causes of famine and poverty in several sub-Saharan countries [3, 4]. Both vector control and the drug administration for preventive or curative therapy are currently used for the control of AAT. The drugs currently used for the treatment of trypanosome infections in animals are not very efficient due to the increasing number of drug resistant strains in several African countries [5] including Cameroon [6]. For vector control, several tools have been developed (the trapping, the use of insecticides and the sterile insect technique) (see [7] for a review). However, the implementation and the field sustainability of these tools remain a challenge. To improve vector control, investigations on endosymbionts which could be implicated in the vector competence of tsetse flies have been undertaken in different tsetse species.

Indeed, besides the parasite, tsetse flies harbor three species of symbiotic bacteria which show different levels of relation with their host [8]. *Wiggleworthia glossinidia*, which is the obligatory symbiont, provides food supplements to maintain the fecundity of the tsetse fly and is therefore important for their larval development and contributes later to the maturation of the immune system [9]. *Sodalis glossinidius*, a maternally transmitted endosymbiont was suspected to be involved in tsetse fly vector competence by favoring parasite installation in the insect midgut [10] through a complex biochemical mechanism involving the production of N-acetyl glucosamine [11–13].

Bacteria belonging to the genus *Wolbachia* are a group of intracellular alpha proteobacteria (order Rickettsiales) that are maternally inherited and occur in numerous arthropod (65% of insects) and filarial nematode species [14]. This bacteria is associated with the reproductive tissues and causes reproductive abnormalities such as cytoplasmic incompatibility (CI), parthenogenesis, male death and feminization [15]. The ability to induce reproductive phenotypes allows them to spread effectively and quickly in host populations [16]. Experimental studies conducted by Geiger et al. [17, 18] showed an association between the presence of symbionts and the ability of tsetse flies to harbor trypanosomes. Moreover, a positive association between the presence of *S. glossinidius* in *G. p. palpalis* and trypanosome infections has been observed in the sleeping sickness foci of Campo and Bipindi in Cameroon [10, 19]. Despite these interesting data, there is little information in the relationship between the presence of endosymbionts and trypanosome infections in other tsetse fly species.

The aim of this study was to determine the prevalence of different trypanosome species and two endosymbionts (*S. glossinidius* and *Wolbachia* sp.) in two tsetse species of two AAT endemic villages of the Adamawa region of Cameroon, in order to evaluate the association between the trypanosome infections and the presence of symbionts in these tsetse species. The study is a preliminary stage of the IAEA-CRP D42015 program that aims to enhance tsetse fly refractoriness to trypanosome infection.

## Methods

### Study site

**Fig. 1:**
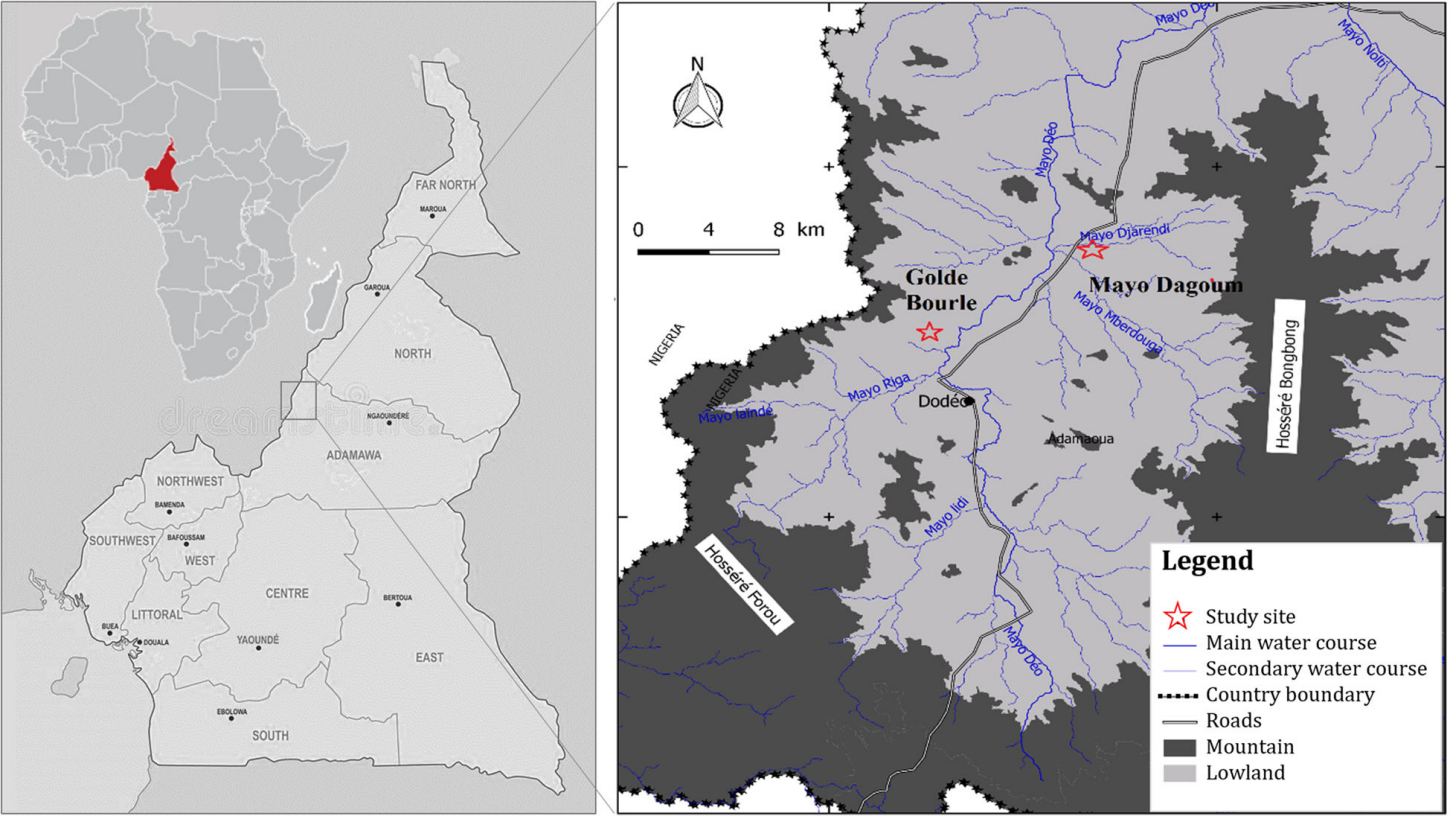
Sampling sites in the Animal African Trypanosomiasis focus of Dodeo

This study was carried out in the villages of Mayo Dagoum (longitude 07°33.06′ and latitude 12°07.11′) and Golde Bourle (longitude 07°33.16′ and latitude 12°07.25′) of the “Faro and Déo” Division in Adamawa region (Fig. 1). The Adamawa plateau covers more than 72, 000 km^2^ with very suitable environments for intensive cattle rearing. Trypanosomiasis is the principal reason of bovine veterinary consultation [20]. The “Faro and Déo” Division is located in northern part of the Adamawa plateau and covers about 11, 000 km^2^ with an altitude of 1000 to 1100 m. The climate is of Sudano-Sahelian type with an average rainfall of 1800 mm [21]. This area has a dense hydrographic network and during the year offers pastures for livestock. However, the pastoral resources are not well exploited because the presence of tsetse flies and the high rate (61.1%) of trypanosome infections in cattle [22] are major constraints to agro pastoral activities.

### Sampling and dissection of tsetse flies

For this study, entomological surveys were conducted in January 2013 at Mayo Dagoum and Golde Bourle villages. During these surveys, 86 traps were put in suitable tsetse fly biotopes for five consecutive days. The geographical coordinates of each trap were recorded using a global positioning system (GPS) device (Garmin Etrex). Tsetse flies were collected twice a day at 11 am and 4 pm to obtain fresh samples for dissection. Species and sex were identified according to morphological criteria [23]. Young flies that had never taken a blood meal, called teneral flies, characterized by the slack aspect of their thorax were excluded from the sample [24]. The non-teneral flies were dissected under a binocular magnifying glass in a drop of sterile 0.9% saline solution. Dissection tools were carefully cleaned successively in 0.5 N NaOH and distilled water after the dissection of each fly to prevent contamination. Tsetse fly midguts were examined under the light microscope at magnification 10X for trypanosome detection and 40X for the confirmation. Midguts and other body parts were separately transferred into Eppendorf tubes containing ethanol (95°). During field manipulations, the tubes were maintained at room temperature; and in the laboratory, they were stored at − 20 °C until use.

### DNA extraction

Frozen samples were thawed and air-dried for 90 min. DNA of tsetse flies’ mid-guts was extracted using cethyl trimethyl ammonium bromide (CTAB) according to the protocol described by Maniatis et al. [25] and Navajas et al. [26]. The mid-guts of the tsetse flies were homogenized with a pestle in a CTAB buffer (CTAB 5%; 1 M Tris, pH 8; 0.5 M EDTA pH 8; 5 M NaCl, 20 μL of β-mercaptoethanol and sterile water) and incubated at 60 °C for 30 min. The DNA was extracted from the lysis mixture with chloroform/isoamylic alcohol (24/1; *V*/V) and precipitated by adding isopropanol (*V*/V). After centrifugation (10,000 g × 10 min), the pellet was rinsed with 70% ethanol, air-dried, and re-suspended in distilled sterile water (20 μL). A quantity of 2 μL of this extracted DNA were launched on 1.5% agarose gel electrophoresis to check the quality of the extracts. The DNA samples were stored at − 20 °C until PCR amplification.

### Confirmation of tsetse fly species

A diagnostic PCR method based on the length polymorphism of ITS1 (*internal transcribed spacer* 1) fragments between tsetse species was performed to confirm species identification [27]. PCR was used to amplify sequences of ITS1 using the primer pairs given in Table 1. The PCR mix contained 6.45 μL double distilled water, 1.5 μL 10X PCR buffer (Bioline), 0.5 μL of 10 mM dNTP, 1 μL of each 10 pmol primer, 1.5 μL of 50 mM MgCl2 (final concentration 2.5 mM), 0.5 U (0.05 μL) of BIOTaq DNA polymerase. A quantity of 3 μL of 1/10e dilution of template extracted DNA was added to PCR mix. Temperature cycles: 5 min 94 °C, 30 cycles of 94 °C for 30 s, 58 °C for 30 s and 72 °C for 1 min, then 72 °C for 10 min.

**Table 1:** Primers used for PCR amplification of trypanosome and bacteria DNA

Specificity	Primer sequence (5′— 3′)	Name	Amplified product (bp)	References
*Sodalis glossinidius*	TGAAGTTGGGAATGTCG AGTTGTAGCACAGCGTGTA	pSG2	120	(Darby et al., 2005) [32]
*Wolbachia.* sp	YATACCTATTCGAAGGGATAG AGCTTCGAGTGAAACCAATTC	16S rRNA	438	(Werren and Windsor, 2000) [34]
	GTCCAATARSTGATGARGAAAC CYGCACCAAYAGYRCTRTAAA	wsp	513	(Baldo et al., 2006) [33]
*T. congolense* (savannah type)	TCGAGCGAGAACGGGCACTTTGCGA ATTAGGGACAAACAAATCCCGCACA	TcS	341	(Moser et al., 1989) [28]
*T. congolense* (forest type)	GGACACGCCAGAAGGTACTT GTTCTCGCACCAAATCCAAC	TcF	350	(Masiga et al., 1992) [29]
*T. brucei sl*	CGAATGAATATTAAACAATGCGCAG AGAACCATTTATTAGCTTTGTTGC	Tbr	164	(Masiga et al., 1992) [29]
*T. vivax*	CTGAGTGCTCCATGTGCCAC CCACCAGAACACCAACCTGA	Tvw	150	(Masiga et al., 1992*)* [29]
*Diag ITS1*	TGGACTTCGGATTAAGTACAACA TCATTATGCGCTATTAAGGTAAGC	Diag	150–400	(Dyer et al., 2008) [27]

### Molecular identification of different trypanosomes

PCR amplifications of animal trypanosomes contained in midguts were performed as described by Moser et al. [28], Masiga et al. [29], Majiwa et al. [30] and Herder et al. [31]. In general, a denaturing step at 94 °C for 5 min was followed by 40 amplification cycles. Each cycle included a denaturing step at 94 °C for 30 s, an annealing step at 58 °C (*T. brucei* sensu lato) or 60 °C (*T. congolense* “forest” and “savannah” types, *T. vivax*) for 30 s and an extension step at 72 °C for 1 min. A final extension was performed at 72 °C for 10 min.

### Molecular identification of *S. glossinidius* and *Wolbachia* sp.

*S. glossinidius* identification was done according to the protocol described by Darby et al. [32] and *Wolbachia* sp. was identified following the method of Baldo et al. [33] and Werren and Windsor [34] methods (Table 1). For PCR amplification of *S. glossinidius*, a denaturing step at 94 °C for 5 min was followed by 40 amplification cycles. Each cycle included a denaturing step at 94 °C for 30 s, an annealing step at 50 °C for 30 s and an extension step at 72 °C for 1 min. A final extension was performed at 72 °C for 10 min. For PCR amplification of *Wolbachia* sp. only the number of cycles (37) and annealing step (59 °C for 45 s) differed from the *Sodalis* PCR protocol.

The amplified products were separated on a 2% agarose gel and visualized under UV illumination. Positive controls corresponding to PCR reagents with reference DNA of trypanosome or symbiont species and negative controls (PCR reagent without any DNA) were included in each set of PCR amplification experiments.

### Expression of results and statistical analysis

Entomological data were expressed in terms of abundance of tsetse flies, estimated by fly apparent density per trap per day (ADT) according to the following expression: ADT = Nc/TD (where Nc is the number of captured tsetse flies, T, the number of traps and D the number of trapping days.

Trypanosomes and symbionts hosted by tsetse flies were expressed in terms of parasite infection rate and symbiont occurrence rate. The Pearson’s chi-square test (χ^2^) was used to compare infection rates between villages [35]. A logistic regression model [36] was used to analyse the association between the presence of symbionts (*S. glossinidius* and *Wolbachia* sp.) and trypanosome infections in each tsetse fly species. All the statistical tests were performed with the software *PASW Statistics* 18 (SPSS Inc., Chicago, IL, USA), and the level of significance was fixed at 0.05.

## Results

### Entomological surveys

A total of 446 tsetse flies belonging to three species or subspecies were caught: 402 (90.1%) *Glossina tachinoides*, 42 (9.4%) *Glossina morsitans submorsitans* and only 2 (0.5%) *Glossina fuscipes fuscipes*. This morphological identification was confirmed by the amplification of ITS1 sequence that revealed a DNA fragment of 221 bp for *G. tachinoides* and a fragment of less than 221 bp for *G. m. submorsitans*.

The mean apparent density (ADT) was 1.037. Its values were 0.785 at Mayo-Dagoum and 1.501 at Golde Bourle (Table 2). Of the 446 flies captured, 30 (6.72%) were teneral and 102 were dead/dessicated. For the remaining 314 flies, 270 *G. tachinoides* and 42 *G. m. submorsitans* were dissected while the two *G. f. fuscipes* samples were not, and were excluded from further analyses, because of the small size of this species. Microscopy enabled the identification of midgut infections of trypanosomes in two *G. m. submorsitans* and one *G. tachinoides*, giving an infection rate of 0.95% (Table 2).

**Table 2:** Results of the entomological survey

Focus	Survey villages	No. traps	No. captured flies	ADT	No. teneral flies (%)	No. dissected flies	No. infected flies (light microscopy) (%)
Dodéo	Mayo-Dagoum	56	220	0.785	17 (7.72)	126	2 (1.58)
	Golde Bourle	30	226	1.501	13 (5.75)	186	1 (0.52)
	Total	86	446	1.037	30 (6.72)	312	3 (0.95)

*No*. number of, *ADT* apparent density per trap per day

### Molecular identification of different trypanosomes

Of the 270 midguts of *G. tachinoides* analyzed, 94 (34.81%) were positive with at least one trypanosome species; 72 (44.7%) trypanosome infections in flies of Golde Bourle and 22 (20.2%) in flies of Mayo-Dagoum. A significant difference (χ^2^ = 17.243; *p* < 0.0001) in the global infection rate was found between the two villages. The numbers of tsetse flies infected by *T. brucei* s.l.*, T. congolense* “forest type” and *T. congolense* “savannah type” were respectively 30 (11.1%), 7 (2.6%) and 37 (13.7%). Trypanosome infection rates were similar between Golde Bourle and Mayo Dagoum for *T. brucei* and *T. congolense* “forest type” but different for *T. congolense* “savannah type” (*p* = 0.003). No *T. vivax* infection was identified. When looking at the type of infection (single or mixed), 74 (27.40%) flies carried single infections and 20 (7.40%) carried mixed infections including 18 double infections (13 TcS/TcF, TcS/Tb sl and TcF/Tb sl) and 3 triple infections (TcS/TcF /Tb sl) (Table 3). The rates of single infections (18.3% versus 33.5%) and mixed infections (1.8% versus 11.2%) significantly differed between Mayo Dagoum and Golde Bourle (*p* = 0.006 and *p* = 0.004) respectively (Table 3).

**Table 3:** Trypanosome infection rates and symbiont occurrence rates in *Glossina tachinoides*

Villages	No. samples	S. g. (%)	W. sp. (%)	Tb s.l. (%)	TcF (%)	TcS (%)	Simple infections (%)	TcS/TcF (%)	TcS/ Tb s.l. (%)	TcF/ Tb s.l. (%)	TcS/TcF/ Tb s.l. (%)	Mixed-infections (%)	Global infection (%)
Mayo-Dagoum	109	50 (45.9)	75 (68.8)	14 (12.8)	1 (0.9)	5 (4.6)	20 (18.3)	0 (0.0)	0 (0.0)	0 (0.0)	2 (1.8)	2 (1.8)	22 (20.2)
Golde Bourle	161	50 (31.1)	109 (67.7)	16 (9.9)	6 (4.1)	32 (19.9)	54 (33.5)	13 (8.1)	3 (1.7)	1 (0.6)	1 (0.6)	18 (11.2)	72 (44.7)
Total	270	100 (37.0)	184 (68.1)	30 (11.1)	7 (2.6)	37 (13.7)	74 (27.4)	13 (4.8)	3 (1.1)	1 (0.4)	3 (1.1)	20 (7.4)	94 (34.8)
Chi-square	/	6.118	0.037	0.556	Fisher exact test	12.85	7.540	9.246	Fisher exact test	Fisher exact test	Fisher exact test	8.276	17.243
*p*-value	/	0.013	0.848	0.456	0.247	0.003	0.006	0.002	0.275	1.000	0.567	0.004	< 0.0001

*No.* number of, *S. g. Sodalis glossinidius*, *W. sp. Wolbachia* sp., *Tb s.l. T. brucei* sensus lato, *TcF T. congolense* forest type, *TcS T. congolense* savannah type

Of the 42 midguts of *G. m. submorsitans,* 17 (40.5%) were infected with at least one trypanosome species: 3 (18.8%) in flies of Mayo Dagoum and 14 (53.8%) in tsetse of Golde Bourle. The global infection rates differed significantly between the two villages (*p* < 0.05). The rates of single infections differed significantly between Mayo Dagoum (6.3%) and Golde Bourle (46.2%) (*p* < 0.01). The number of flies infected with each trypanosome species or subspecies were 7 (16.7%), 4 (9.5%), 1 (2.4%) and 1 (2.4%), respectively, for *T. brucei* s.l., *T. congolense* “savannah type”, *T. congolense* forest type and *T. vivax*. The differences in these infection rates were not tested between the villages because of the low numbers of the infected flies (Table 4).

**Table 4:** Trypanosome infection rates and symbiont occurrence rates in *Glossina morsitans submorsitans*

Villages	No.	*Sodalis glossinidius* (%)	*Wolbachia* sp. (%)	Tb sl (%)	TcS (%)	TcF (%)	Twx (%)	Simple infections (%)	Mixed infections			Global infection (%)
									TcS/TcF	TcS/Tbr	Tbr/TcF	
Mayo-Dagoum	16	7 (43.8)	11 (68.8)	0 (0.0)	0 (0.0)	0 (0.0)	1 (6.3)	1 (6.3)	1	1	0	3 (18.8)
Golde Bourle	26	5 (19.2)	14 (53.9)	7 (43.8)	4 (15.4)	1 (3.8)	0 (0.0)	12 (46.2)	1	1	0	14 (53.8)
Total	42	12 (28.6)	25 (59.5)	7 (16.7)	4 (9.5)	1 (2.4)	1 (2.4)	13 (31.0)	2	2	0	17 (40.5)

*No.* number of samples, *Tb sl T. brucei* sensus lato, *TcF T. congolense* forest type, *TcS T. congolense* savannah type, *Twx Trypanosoma vivax*

### Molecular identification of symbionts

*S. glossinidius* was identified in 100 (37%) of the 270 *G. tachinoides*: 50 (45.9%) in flies of Mayo Dagoum and 50 (31.1%) in flies of Golde Bourle. Comparing the percentages of flies harboring *S. glossinidius*, a significant difference (χ^2^ = 6.118; *p* = 0.013) was obtained between Mayo Dagoum and Golde Bourle (Table 3). For the 42 *G. m. submorsitans, S. glossinidius* was identified in 12 (28.6%) of them: 7 (43.8%) in Mayo Dagoum and 5 (19.2%) in Golde Bourle (Table 4). These values did not differ between villages (*p* > 0.05).

Of the 270 *G. tachinoides*, *Wolbachia* sp. was identified in 184 (68.1%) of them. Between Mayo Dagoum and Golde Bourle, no significant difference (χ^2^ = 0.037; *p* = 0.848) was found in the percentage of flies harboring *Wolbachia* sp. (Table 3). Of the 42 *G. m. submorsitans, Wolbachia* sp. was identified in 59.5% of them. Comparing the percentage of flies harboring *Wolbachia* sp., no significant difference (*P* > 0.1) was observed between Mayo Dagoum and Golde Bourle (Table 4).

### Relationship between trypanosome infections and the presence of symbionts

Of the 100 *G. tachinoides* harboring *S. glossinidius,* 37 (37%) were infected by at least one trypanosome species (these flies were noticed (S + trp+)) and 63 (63%) were not (S + trp-). Of the 170 *S. glossinidius* negative flies, 58 (34.12%) were infected by trypanosomes (S-trp+) while 112 (65.88%) were not (S-trp-). Of 184 *G. tachinoides* harboring *Wolbachia* sp., 60 (32.6%) were infected by trypanosomes (W + trp+) and 124 (67.4%) were not (W + trp-). For the remaining 86 *G. tachinoides* for which *Wolbachia* sp. was not identified, 32 (37.2%) were infected by trypanosomes (W-trp+). No significant difference was observed between tsetse flies harboring together trypanosome infections and *Wolbachia sp.,* or/and *S. glossinidius* and flies with only trypanosome infections (Table 5).

**Table 5:** Relationship between the presence of *Sodalis glossinidius* and the flies’ infection by trypanosomes in *G. tachinoides*

Combination symbiont/trypanosome	Probability values and odds ratio		
	P	Odds ratio	95% CI
S. vs Tb sl	0.797	0.909	[0.440–1.877]
W. vs Tb sl	0.075	2.199	[0.925–5.228]
S. vs TcF	0.694	0.837	[0.345–2.032]
W. vs TcF	0.768	1.149	[0.458–2.883]
S. vs TcS	0.053	1.798	[0.991–3.262]
W. vs TcS	0.182	0.659	[0.358–1.215]
S. vs All trypanosomes	0.563	1.164	[0.695–1.950]
W. vs All trypanosomes	0.572	0.858	[0.503–1.462]
Symbionts (S. + W.) vs All trypanosomes	0.942	1.022	[0.561–1.863]

*S. S. glossinidius*, *W. Wolbachia* sp., *vs* versus, *Tb sl. T. brucei* s.l., *TcF T. congolense* forest type, *TcS T. congolense* savannah type

## Discussion

### Tsetse flies trapping

The entomological survey has revealed the presence of three tsetse fly species whereas previous studies detected only two taxa (*G. tachinoides* and *G. m. submorsitans*) in forest galleries and pasture bordering the watercourse in the Adamawa savannah [21]. Moreover, the high frequency of *G. tachinoides* compared to *G. m. submorsitans* suggests a modification of the distribution compared to previous results [37, 38] that reported high densities of *G. m. submorsitans* in the Adamawa plateau. This discrepancy may be explained by both environmental and climatic changes that have induced high or low harmful effects according to tsetse fly species, and the development of agriculture zones. The high density of tsetse flies at Golde Bourle (1.506) compared to Mayo-Dagoum (0.785) can be explained by the presence of a forest gallery with diverse wild animals along the great river of Golde Bourle which has created favorable microenvironment for tsetse flies.

### Trypanosome infection rate

Trypanosome infection rates were relatively low, varying from 2.4 to 16.7% in *G. m. submorsitans* and from 2.6 to 13.7% in *G. tachinoides*, as compared to values reported in *G. m. submorsitans* (1.5 to 36.4%) and *G. tachinoides* (2.3 to 20.7%) in Yankari National Park and Wuya areas (Nigeria) by Isaac et al. [39]. *T. congolense* was the predominant species, confirming the high transmission of that parasite in “Faro and Déo” as previously observed by Mamoudou et al. [6, 40] and Tanembe et al. [22] when studying cattle trypanosomes and the main parasites responsible for AAT in livestock. However, the use of species-specific primers could not reveal all the trypanosome species and might have lowered the infection risk. The association of DNA sequencing of Glyceraldehyde 3-Phosphate Dehydrogenase and the Fluorescent Fragment Length Barcoding methods would have been useful. The low prevalence of *T. vivax* could be due to the fact that DNA was extracted only from the midguts.

### Symbiont occurrence rates

In *G. tachinoides*, the mean occurrence rate of *S. glossinidius* (37%) is low compared to 54.9% revealed in *G. p. palpalis* of Bipindi and Campo in the Southern Cameroon. However, this value is higher than 3.7 and 16% reported, respectively, for *G. austeni* and *G. pallidipes* in Shimba Hills National Reserve in Kenya [41]. The differences observed between these studies may be due to the diversity of tsetse species and the occurrence of different genotypes of symbionts [18, 19].

The high prevalence of *Wolbachia* sp. in *G. tachinoides* (68.1%) and in *G. m. submorsitans* (58.5%) does not corroborate results of Doudoumis et al. [42] who reported the absence of *Wolbachia* in tsetse flies of the *G. palpalis* group. However, the species *G. m*. *submorsitans* was not included in their study. These observations suggest that the prevalence of *Wolbachia* sp. may depend on the ecological conditions of tsetse fly populations [43, 44].

### *Sodalis* and *Trypanosoma* sp. relationships

In the present study, no significant association was found between the presence of symbionts and trypanosome infections in *G. tachinoides*. The lack of association may be due to several reasons: firstly, the low trypanosome infection rate observed in tsetse flies (2.5 to 13.7%) compared to 9.1 to 15.6% in South Cameroon was not a favorable condition for the association tests; secondly, tsetse fly species might differ in whether *Sodalis* affects them (*G. p. palpalis* versus *G. tachinoides*); the association may also vary according to specific genotypes of *S. glossinidius* rather than the presence or absence of the symbiont as shown by Geiger et al. [18].

*S. glossinidius* is suspected to be involved in the vector competence of *Glossina* by favoring parasite installation in the insect midgut through a complex biochemical mechanism involving the production of N-acetyl glucosamine [11, 12]. This sugar was reported to inhibit tsetse-midgut lectin lethal for the procyclic forms of the trypanosome [12, 13]. The presence of *S. glossinidius* would thus allow the trypanosome to establish in the midgut [12, 45, 46]. The studies conducted by Geiger et al. [17, 18] supported this hypothesis but did not consider it as an absolute condition. Recently, Farikou et al. [10, 19] observed a positive association between the presence of *S. glossinidius* and the rate of some trypanosome infections in Campo and Bipindi HAT foci in South Cameroon. These results suggested that *S. glossinidius* could favor establishment of trypanosome infections in *G. p. palpalis*. The identification of *S. glossinidius* genotypes occurring in these flies would allow to better understand these relations.

### *Wolbachia* sp. and *Trypanosoma* sp. relationships

More than 50% of tsetse flies harbored *Wolbachia* sp. among which 26% of *G. tachinoides* and 23% of *G. m. submorsitans* were infected by trypanosomes, but no association test was significant. These tests should be performed with different haplotypes of *Wolbachia* sp. revealed by genotyping and sequencing of the alleles identified, to allow a better exploration of the possible existence of relationships between the presence of *Wolbachia* sp. and the level of trypanosome infection.

## Conclusions

This study revealed the presence of three species of tsetse flies dominated by *G. tachinoides*. Some tsetse flies harbored symbionts (*Sodalis glossinidius* and/or *Wolbachia* sp.) and animal trypanosomes, but no association between the presence of symbionts and the level of trypanosome infection was significant. Additional studies searching for associations between the presence of some symbiont haplotypes and trypanosome infections are required to better elucidate tsetse fly/symbiont/trypanosome relations.

## Abbreviations

AAT: Animal African trypanosomosis
ADT: Apparent density per trap per day
CTAB: Cethyl trimethyl ammonium bromide
DNA: Deoxyribonucleic acid
GPS: Global positioning system
HAT: Human African trypanosomosis
ITS: Internal transcribed spacer
MSEG: Special Mission for Tsetse Eradication
NaOH: Sodium hydroxide
PCR: Polymerase chain reaction
Tb: *Trypanosoma brucei*
Tcf: *Trypanosoma congolense* forest type
Tcs: *Trypanosoma congolense* savannah type
Trp: *Trypanosoma*
